# MPscore: A Novel Predictive and Prognostic Scoring for Progressive Meningioma

**DOI:** 10.3390/cancers13051113

**Published:** 2021-03-05

**Authors:** Feili Liu, Jin Qian, Chenkai Ma

**Affiliations:** 1 Department of Neurosurgery, Huashan Hospital, Shanghai Medical College, Fudan University, Shanghai 200040, China; liufeili@huashan.org.cn; 2 Neurosurgical Institute, Fudan University, Shanghai 200040, China; 3 Shanghai Clinical Medical Center of Neurosurgery, Shanghai 200040, China; 4 Shanghai Key Laboratory of Brain Function and Restoration and Neural Regeneration, Shanghai 200040, China; 5 Department of Radiation Oncology, Stanford University, Stanford, CA 94305, USA; jinqian@stanford.edu; 6 Department of Surgery, the University of Melbourne, Melbourne 3050, Australia

**Keywords:** meningioma, consensus clustering, subtype, ALPL, immune cells, transcriptome, random forest, progression

## Abstract

**Simple Summary:**

Subtyping for meningioma is urgently required to stratify the patients with high risks of recurrence and progression due to the intertumoral heterogeneity in meningioma. Here, we performed a consensus clustering of 179 meningiomas and identified progressive subtype (subtype 3) based the transcriptome profiles. Loss of chromosome 1q along with Neurofibromin 2 (NF2) mutation or loss of chromosome 22p is exclusively presented in subtype 3 meningioma. DNA methylation analyses of meningioma subtypes also suggested hypermethylation was observed in subtype 3 meningioma. Our findings identified low expression of Alkaline Phosphatase (ALPL) is the most significant feature in progressive subtype of meningioma. We constructed and validated a meningioma progression score (MPscore) to characterize the progressive phenotype in meningioma. The predictive accuracy has also been validated in three independent cohorts. Therefore, MPscore can be potentially useful for meningioma recurrence prediction and stratification.

**Abstract:**

Meningioma is the most common tumor in central nervous system (CNS). Although most cases of meningioma are benign (WHO grade I) and curable by surgical resection, a few tumors remain diagnostically and therapeutically challenging due to the frequent recurrence and progression. The heterogeneity of meningioma revealed by DNA methylation profiling suggests the demand of subtyping for meningioma. Therefore, we performed a clustering analyses to characterize the progressive features of meningioma and constructed a meningioma progression score to predict the risk of the recurrence. A total of 179 meningioma transcriptome from RNA sequencing was included for progression subtype clustering. Four biologically distinct subtypes (subtype 1, subtype 2, subtype 3 and subtype 4) were identified. Copy number alternation and genomewide DNA methylation of each subtype was also characterized. Immune cell infiltration was examined by the microenvironment cell populations counter. All anaplastic meningiomas (7/7) and most atypical meningiomas (24/32) are enriched in subtype 3 while no WHO II or III meningioma presents in subtype 1, suggesting subtype 3 meningioma is a progressive subtype. Stemness index and immune response are also heterogeneous across four subtypes. Monocytic lineage is the most immune cell type in all meningiomas, except for subtype 1. CD8 positive T cells are predominantly observed in subtype 3. To extend the clinical utility of progressive meningioma subtyping, we constructed the meningioma progression score (MPscore) by the signature genes in subtype 3. The predictive accuracy and prognostic capacity of MPscore has also been validated in three independent cohort. Our study uncovers four biologically distinct subtypes in meningioma and the MPscore is potentially helpful in the recurrence risk prediction and response to treatments stratification in meningioma.

## 1. Introduction

Meningioma is the most common primary tumor in the central nervous system with mesodermal-arachnoid origin. Around 80% of meningiomas are benign and curable by surgical resection alone. However, 20% of meningioma will recur after initial surgical operations and a further comprehensive treatment regimen is required [1]. Currently, the histoimmunochemistry-based WHO pathology grade system is the main predictor for patient outcomes with meningioma. WHO grade I meningiomas are usually defined as “benign” tumors while grade II (atypical) and grade III (anaplastic) meningiomas are more aggressive and to recur [2]. Meningiomas of high grade (grade II and III) are considered as having higher risk of recurrence than that in grade I meningiomas. Although the 2016 WHO classification of central nervous system tumors histologically subdivides meningioma into 15 subtypes, it does not indicate the prognosis of patients precisely [3]. To precisely diagnose meningioma and complement the grade system, a few investigations on the genetics and epigenetics of meningioma have been performed recently.

Precise cancer diagnoses are critical for the suitable treatment strategy for cancer patients, therefore, a few studies were under investigation on the biological biomarkers for meningioma. Neurofibromin 2 (NF2) mutation is the first characterized alteration in meningioma and is observed in approximately 80% of high grade meningiomas, suggesting that it is potentially a prognostic biomarker. Structural variants including loss of chromosomes 6q, 9p, 10q, 14, and 18q, as well as gains in 17q and 20q are also associated with recurrence of meningioma [4,5]. Gene expression signature shows its association of patient survivals and another panel of two gene (PTTG1 and LEPR) expressions demonstrates association with the recurrent meningioma [6,7,8]. More recently, a DNA methylation-based classification defining six distinct subtypes with clinical relevance reveals a stronger association of clinical outcomes than conventional WHO classification. Unsupervised clustering of DNA methylation profiles in meningiomas identifies two distinct subgroups associated with distinct recurrence-free survival [9]. Similarly, Sahm et al. also identified two major and six minor subgroups of meningioma from DNA methylation profile with significantly different clinical behaviors and non-NF2 mutated meningiomas are clustered in one “benign” subgroup [10]. In addition, a recent study displays a 64-CpG loci-based predictor that indicates the risk of meningioma recurrence [11]. Overall, these studies highlighted the significance of biological biomarkers for the purpose of precise diagnosis and individualized management of meningioma.

The consensus clustering of cancer transcriptomes yields a robust and precise diagnosis for heterogeneous tumors, especially in brain tumors. Glioblastoma multiforme, the most malignant glioma, is reclassified into four subtypes with distinct transcriptome and clinical outcomes [12]. Similarly, children with wingless (WNT) subtype of medulloblastoma have the most favorable outcomes than children with others subtypes of medulloblastoma [13]. In addition, patients with posterior fossa group A or supratentorial REL-associated protein (RELA)-positive ependymoma show dismal prognosis of all subtypes of ependymoma and the risk stratification by comprehensive molecular subgrouping is superior to histological grading [14]. These subtyping of heterogeneous tumors shed a light on precision diagnosis and treatment in cancer patients. However, whether meningioma could be robustly clustered into subtypes by transcriptomes with clinical disperse behaviors remains unclear.

Immunotherapy is recently emerging as a new hope for cancer treatment and management. As vasculature is enriched in meningioma, peripheral and central system immune cells are present in meningioma, which enable the application of immunotherapy. Exclusion of cytotoxic CD8+ T cells and CD4+ helper T cells are observed in high-grade meningioma, suggesting the enhanced T cells therapy is potentially treatment for recurrent meningioma [15]. The abundance of PD-L1 expression in meningioma also suggests that meningioma patients likely benefit from the immune checkpoint inhibition [16]. Therefore, a few clinical trials are under investigation to explore the efficacy of checkpoint inhibition in high-grade and/or recurrent meningioma [17]. Despite that, we are still not clear whether the immune infiltration has shared the same pattern across all types of meningioma.

In this study, we consolidated the total RNA sequencing results from 179 WHO I, II or III meningiomas and identified four biologically distinct subtypes of meningioma using consensus clustering. These subtypes of meningioma also have mutually different frequency of DNA methylation pattern, gene fusion and infiltrating immune cell profiles. Further, our results of random-forest-based machine learning identified Alkaline Phosphatase (ALPL) is the feature gene in the most aggressive subtype of meningioma. We established and validated Meningioma Progression score (MPscore) to characterize the risk of progression in meningioma. Our subclassification provides a further insight on the understanding of biological behaviors in meningioma.

## 2. Materials and Methods

### 2.1. Data Preprocessing and Tissue Sample Validation

RNA sequencing profiles of 179 meningioma were included in this study [18,19]. The raw reads were preprocessed prior to the transcriptome analysis. Trim-galore was utilized to remove adapters and low-quality reads with the follow parameters (-q 25 --length 50 -e 0.1 --stringency 5). The trimmed reads were subjected to alignment against hg38 by STAR 2.5.3a with default settings. FeatureCounts of Subread 1.6.4 was utilized for quantifying the counts of the RNA transcripts. Batch effects were corrected by removeBatchEffect. The Transcripts per kilobase million (TPM) rate was calculated for normalization and subjected to downstream analyses.

For DNA methylation array datasets, a cohort of 39 meningiomas from a previous study was utilized for the investigation of DNA methylation alteration between subtypes [20]. Raw signals were quantile-normalized before the removal of the probes located in sexual chromosomes and the single nucleotide polymorphisms (SNP). The crossing-reactive probes were also removed before the downstream analyses. The M values were used for statistical analyses while the beta values were used for the data visualization and biological interpretation. We utilized the manifest “IlluminaHumanMethylation450kanno.ilmn12.hg19” for the CpG probes annotation.

A small cohort of six meningioma samples along with two independent datasets were utilized for the meningioma progression scoring validation. The detailed methods were described in the supplementary methods.

### 2.2. Consensus Clustering

Principal component analysis (PCA) was employed for dimension reduction exploration. The distances of samples were determined by the root-mean-square deviation (Euclidean distance) of the top 2000 genes. Hierarchical clustering with agglomerative average linkage was performed in this study, as our basis for consensus clustering, to detect the robust clusters. The distance metric 1-(Pearson’s correlation coefficient) was used for variances detection between samples. SigClust was performed to establish the significance of the clusters in a pairwise fashion. All subtype identification was performed by the package “ConsensusClusterPlus”.

### 2.3. Differentially Expressed Gene (DEG) Analysis

Differentially expressed gene (DEG) analysis was performed for identification of featured genes in each subtype. DEG was performed on linear modelling of indicated (co-) variates on expression values by limma (Ritchie et al. 2015). *p*-values generated from limma modelling were corrected for multiple hypothesis testing by Benjamini and Hochberg false discovery rate (FDR) adjustments. Each subtype was tested against all other groups to generate this subtype featured genes. The FDR-adjusted *p*-values < 0.05 and |log Fold Change (FC)| > 2 were considered statistically significant. The DEGs between subtypes were visualized by heatmaps.

### 2.4. Stemness Index (SI) Prediction

A stemness index (SI) model utilizing an OCLR algorithm on pluripotent stem cells was generated by Malta et al. to predict the proportion of stem cells per given cancer sample [21]. We applied this stemness index model to the 179 meningiomas using Spearman correlation for RNA expressions. The whole workflow is available from: https://bioinformaticsfmrp.github.io/PanCanStem_Web/ (accessed on 1 August 2020).

### 2.5. Copy Number Alteration (CNA) and DNA Methylation Analysis

The copy number alteration (CNA) was calculated by the package “conumee” using DNA methylation dataset. All chromosomes alterations of each included samples were calculated and plotted by “CNV.genomeplot” with default setting. For structural variation, the chr1p or chr22q loss (mean of chromosomal arm less than 0.1) was selected by a reduction of copy number in chr1q or chr22q [19]. The stemness index was predicted for each meningioma. The CpG island methylation phenotype (CIMP) was defined as that most variable CpG loci (a standard deviation larger than 0.2 in a certain subtype) were hypermethylated. Subtype-specific CpG loci signatures were determined by the package “limma” using M value in a pairwise fashion and the |logFC| > 2 and adjusted *p* value < 0.05 was considered as the statistical significance.

### 2.6. Immune Cell Infiltration Prediction

By applying the Microenvironment Cell Populations-counter (MCPcounter) method, the abundances of eight immune cells infiltrating meningioma were predicted to explore the feasibility of immunotherapy in meningioma [22]. Eight immune cell proportions were compared in each subtype of meningiomas. PD-L1 expression in each subtype of meningiomas was also compared.

### 2.7. Fusion Genes Identification

We employed the STAR-Fusion 1.8.1 against hg38 to detect the fusion genes in meningiomas with default settings. The most common fusions in all and subtypes of meningiomas were compared to uncover the subtype featured fusion. Fusions spanning two mutually different chromosomes were considered as the interchromosomal fusions.

### 2.8. Random Forest Model

The most importance of transcripts in subtype of meningiomas were identified by random forest (RF) using the “randomForest” package with default settings. The cross-validated prediction performance of this model was iterated by the function “rfcv” with 10-fold cross-validation and a removal of 1.5 variables in each step. The discrimination of the most important gene (variable) for meningioma in the given subtype was predicted “pROC” against all other subtypes. The area under the curve (AUC) was utilized here as the accuracy for subtype prediction.

### 2.9. Meningioma Progression Score (MPscore)

The R package “ssGSEA” was utilized to construct the meningioma progression score (MPscore) per given sample using the “GSVA” package [23]. Prior to MPscore construction, we selected DEGs of subtype 3 meningioma as the progressive gene signature and reference. For accurately surrogating the progressive phenotype, we sub-divided the gene list into up- or down-regulated gene lists in subtype 3, calculated the scores through ssGSEA, respectively. The MPscore was the sum of the difference of ssGSEA-predicted scores from up- or downregulated gene list.

MPscore=∑ssGSEA(upregulated signatures)−ssGSEA(downregulated signatures)

### 2.10. Statistical Analysis

R software version 3.5.1 (R Core Team, Vienna, Austria) was used for all statistical analyses. Student’s t test was used for the statistical comparison of two groups. ANOVA was performed to test the statistical significance between more than three groups and Tukey’s honestly significant difference (HSD) test was conducted as a post hoc test when the results of ANOVA indicated significance. A *p* value less than 0.05 was considered statistically significant.

## 3. Results

### 3.1. Transcriptome Profiling Unravels Four Distinct Subtypes in Meningiomas

**Figure 1:**
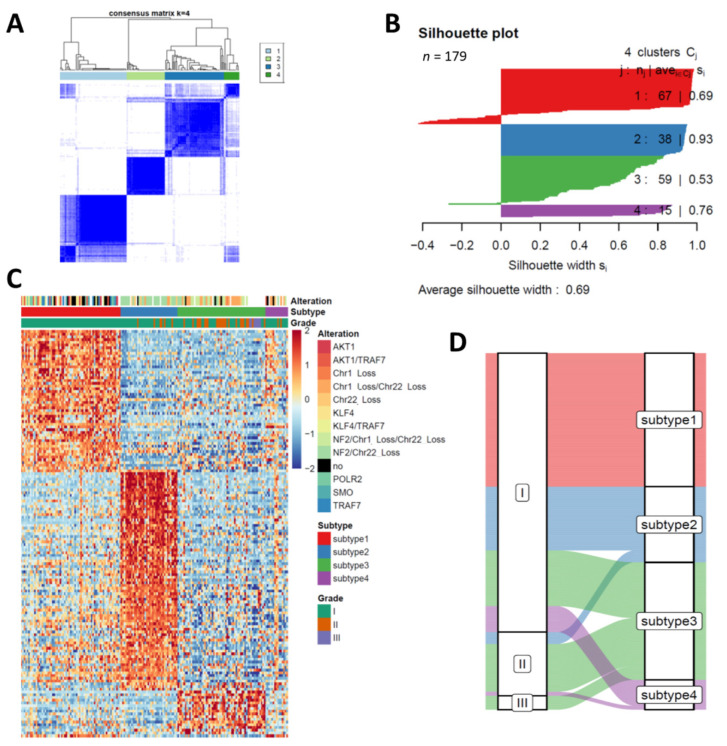
Consensus clustering of 179 meningiomas on gene expression. (**A**) Consensus matrix plot display consensus (k = 4) is the best number for subgrouping. (**B**) Clustering significance of each subtype is indicated by silhouette plot. (**C**) Heatmaps of the upregulated genes in each subtype highlights the heterogeneity of meningioma. Somatic mutation and copy number alteration of each sample is labelled in the top. no, no alteration is detected in given sample. (**D**) Sankey plot displays the change of classification of each sample from WHO grade to transcriptome subtypes.

A total of 179 meningiomas composed from two cohorts was included in this study. Seven of 179 are WHO III meningioma, 32 are WHO II meningioma while the rest are WHO I meningioma. The detailed clinical and pathological information are listed in Table 1. After normalization and batch correction by remove Batch Effect, TPM of gene level count was utilized for all RNA sequencing samples. PCA identified there was a heterogeneity of meningioma that was not caused by the batches on transcriptome level (Figure S1). To explore the heterogeneity of meningiomas, we performed consensus clustering on all 179 samples after integration of all 179 meningiomas in one unified dataset. Consensus average linkage hierarchical clustering of 179 samples identified four robust subtypes with clustering stability increasing for k = 2 to k = 6, but not for k > 4 (Figure 1). Cluster significance was evaluated using SigClust and all pairwise cluster significance tests were statistically significant (Figure S2). Notably, all grade III anaplastic meningiomas (7/7) and most grade II atypical meningiomas (24/32) were clustered in subtype 3, suggesting subtype 3 meningioma was most malignant subtype (Figure 1C and Figure S2). No atypical or anaplastic meningiomas were clustered in subtype 1, suggesting meningiomas in this subtype have mild progression (Figure 1C, Table 1). To further explore the key feature genes in each subtype, we conducted the differentially expressed gene (DEG) analysis of four subtypes. The DEGs of each subtype against all other subtypes was determined by limma package. A total of 263 genes were identified as DEGs between subtypes of meningiomas and four subtypes demonstrated distinct expression pattern of these 263 DEGs (Table S1, Figure 1C). More interestingly, variety of somatic mutations were presented in subtype 1 meningioma, except for NF2 mutation and chromosome 1p and chromosome 22q loss. NF2 mutation with chromosome 22q loss was only detected in subtype 2 while almost all meningiomas containing chromosome 1p loss with NF2 mutation or chromosome 22q loss were clustered in subtype 3 (Figure 1C). These results demonstrated our transcriptome distinct clustering surrogated the genetic variation in meningioma. The Sankey plot revealed the change of meningioma subtypes from WHO classification to the clustering subtype (Figure 1D), suggesting some WHO I meningioma had a similar biological behavior to the high grade meningioma and conventional histopathology-based classification might not surrogate the malignancy of meningioma.

### 3.2. Stemness Indexes Reveal the Different Progressive Potentials between Subtypes

Stemness is regarded as the key factor of carcinogenesis and resistance to chemotherapy. Malta et al. established an index based on the stemness markers indicating the cancer stem cell proportion in the given tumor sample [21]. Although the cancer stem cells are yet to be isolated from meningioma, cancer stem cell and embryonic stem cell markers have been widely identified from meningiomas and strongly associated with patients outcomes [24,25]. To infer the proportion of cancer stem cells between subtypes of meningiomas, we utilized a reference-based matrix to generate stemness indexes (SIs) for each meningioma in our study. There was a significant difference of SIs between low grade and high-grade meningioma (ANOVA, *p* = 0.00195). As expected, the SIs were correlated with the grade of meningiomas and anaplastic meningioma revealed the highest SIs of all grades meningiomas (Figure S3), suggesting SI was likely associated with the malignancy of meningioma. We also observed a remarkable difference of SIs between subtypes of meningiomas and subtype 3 and 4 had higher SIs than the other two subtypes (ANOVA, *p* = 6.79 × 10^−13^), suggesting meningiomas in subtype 3 and 4 are likely to have higher proportion of cancer stem cells. Notably, SI of subtype 3 was not the highest of all subtypes, though subtype 3 is the most progressive subtype, suggesting cancer stem cells were probably not the driven force during meningioma progression. Taken together, our results suggested the malignancy of meningioma rather than meningioma recurrence was associated with high levels of SIs.

### 3.3. Epigenetic Alterations Recapitulate the Subtyping of Meningioma

**Figure 2:**
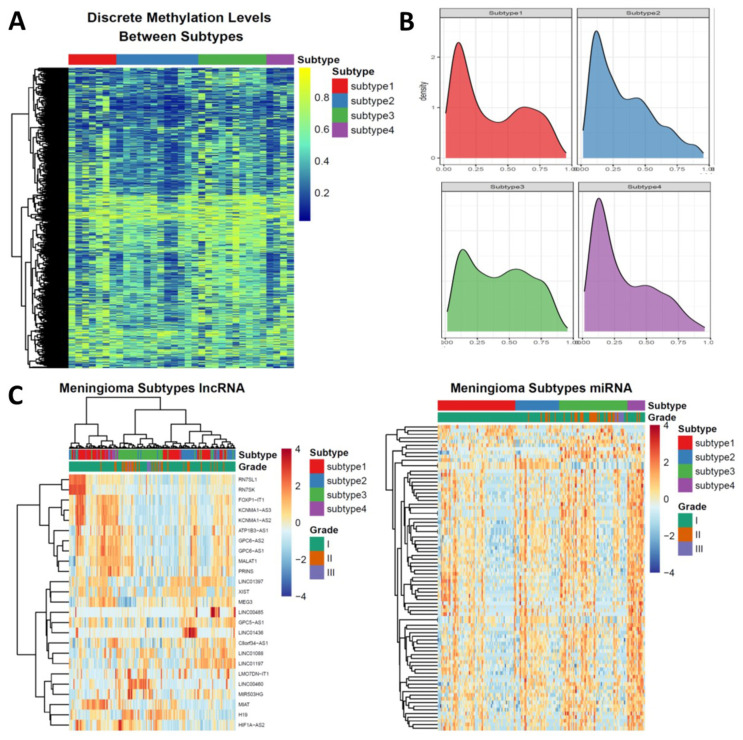
The epigenetic landscape of the subtypes of meningioma. (**A**) Heatmap showing the most variable CpG loci across all subtypes of meningioma. Majority of CpG loci in the subtype 3 meningioma is hypermethylated. (**B**) Density plots showing the methylation levels of subtype specific CpG loci signatures across all subtypes. (**C**) long non-coding RNA (left) and miRNA (right) expression subdivide the subtypes of meningiomas. The differentially expressed long non-coding RNAs or microRNAs were utilized for heatmap clustering.

As distinct DNA methylation levels have been observed in nonrecurrent meningioma but not in recurrent meningioma, we then test whether the subtyping of meningioma could be reflected by DNA methylation. To achieve this, we formed the meningioma cohort of DNA methylation by utilizing a DNA methylation array dataset containing 39 meningioma samples. We first determined the Copy Number Alteration (CNA) of each sample using the package “conumee” because chromosome 1p/22q codeletion and chromosome 22q loss alone were the featured events in the subtype 3 and subtype 2 meningioma, respectively (Figure S4A). Secondly, as we found subtype 4 meningioma has the highest SI of all subtypes, we utilized the DNA methylation version of the same SI prediction tool to predict the SI for the cohorts of DNA methylation profiling. The average SI of subtype 4 meningioma was larger than 0.5 (Figure S3), therefore, the samples with the SIs of higher than 0.5 were considered as the subtype 4 meningioma in DNA. Finally, we assigned the samples having no chromosome 1p or 22q loss into the subtype 1 meningioma from the rest samples of this cohort (Figure S4A). A total of 33 meningioma with subtyping was included in this study and a large variation of the DNA methylation levels across subtypes were observed in the PCA plot (Figure S4B). To further uncover the DNA methylation pattern in each subtype, especially in the subtype 3 meningioma, we then explored whether the CpG island methylation phenotype (CIMP) presented in the subtypes of meningioma. Notably, the most variable CpG loci (with the standard deviation larger than 0.2 of all meningioma) in the subtype 3 meningioma were hypermethylated (Figure 2A), suggesting the association of CIMP with the subtype 3 meningioma. We then hypothesized the hypermethylated CpG loci signatures of the subtype 3 meningioma were located in the promoter areas as we found the majority of DEGs in the subtype 3 were downregulated. To address this, we identified the subtype specific CpG loci signatures by a pairwise approach (Figure S4C). The density plots of DNA methylation levels in the promoter area revealed a hypermethylated CpG loci signature in the subtype 3 meningioma (Figure 2B). In addition, the subtype 3 meningioma had a significantly higher DNA methylation level in the CpG island than the other subtypes (Figure S4D). These results suggested DNA hypermethylation was probably the featured event during the progression of meningioma.

Because long non-coding RNAs and microRNAs are associated with biological status and specific biomarkers in cancers, we calculated the transcript abundance for 2531 lncRNAs and 1715 miRNAs, respectively. A total of 25 lncRNAs were identified as differentially expressed lncRNAs. The cluster of lncRNAs were concordant with mRNA clustering. Unsupervised clustering of these 25 lncRNAs revealed the significant lncRNA expression features in subtype 3. Specifically, RN7SL1, RN7SK, FOXP1-IT1, KCNMA1-AS3, KCNMA1-AS2, ATP1B3-AS1, GPC6-AS2, GPC6-AS1, MALAT1, PRINS, LINC01397, XIST, MEG3, LINC00485, GPC5-AS1, and LINC01436 were downregulated in subtype 3 while the expression of LMO7DN-IT1, LINC00460, MIAT, H19, and HIF1A-AS2 was higher than these in other subtypes (Table S2, Figure 2C). For miRNAs, unsupervised clustering of differentially expressed miRNAs subdivided meningiomas. In subtype 1, subtype 2 and subtype 3, each subtype could be subdivided into two subgroups based on miRNAs expression (Table S2, Figure 2C). These results suggested ncRNAs played critical roles in distinct subtypes of meningiomas.

### 3.4. Novel Fusion Genes were Identified between Subtypes

**Figure 3:**
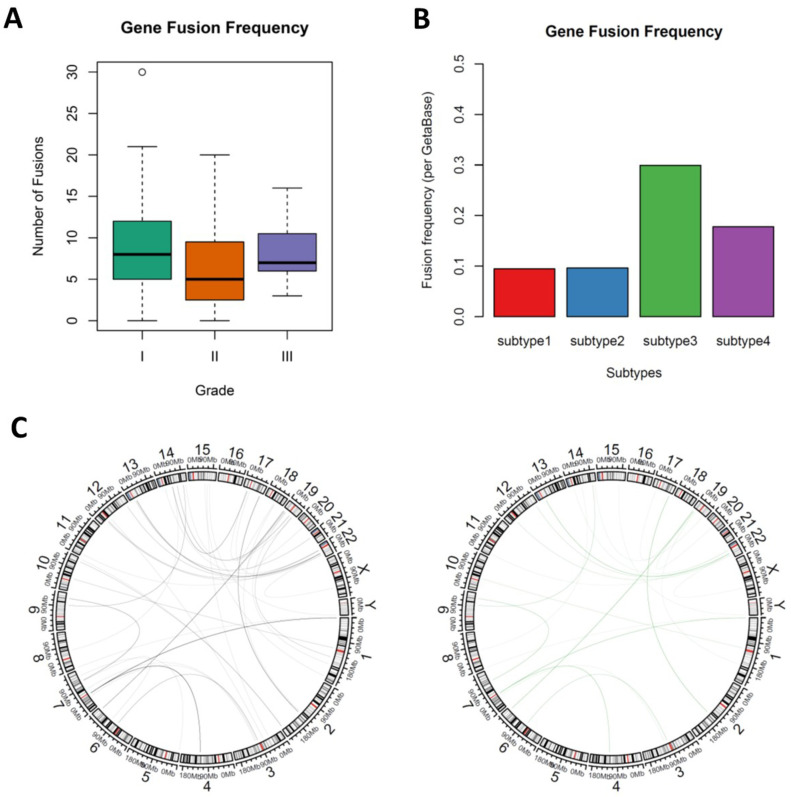
The landscape of fusion genes in meningiomas. (**A**) Gene fusion frequency between WHO grades; right, ANOVA test, *p* = 0.115. (**B**) Bar plot showing the interchromosomal gene fusion frequency of each subtypes. (**C**) Circus plots showing the interchromosomal gene fusion in all meningioma (left) and subtype 3 meningioma (right).

A small cohort uncovered NF2 gene fusion is strongly associated with meningioma progression [26], however, a comprehensive landscape of gene fusions in meningioma and whether subtypes of meningiomas had distinct gene fusions features were still unclear. We utilized STAR-fusion 1.8.1 to investigate the novel gene fusions between subtypes of meningiomas. Grade I and subtype 1 meningiomas had higher fusion frequency than higher grade or other subtype meningiomas, though the fusion frequencies were not significantly different between subtypes (ANOVA, *p* = 0.115) and grades (ANOVA, *p* = 0.13), which was consistent with previous findings (Figure 3A and Figure S5). The most common gene fusion in this study is RP11-1102P16.1--EYA1 (195/1489, 13.10%), followed by RP11-680G10.1--GSE1 (148/1489, 9.94%) and CTC-786C10.1--RP11-680G10.1 (124/1489, 8.33%), which of all were yet to be fully described before. The most frequent fusion was RP11-1102P16.1--EYA1 (101), MIR100HG--RP11-166D19.1 (33), RP11-680G10.1--GSE1 (49) and RP11-1102P16.1--EYA1 (21) for subtype 1, subtype 2, subtype 3 and subtype 4, respectively (Table S3). In addition, we also identified novel NF2-related fusions: NF2--LUZP4 (chromosomes 22q and chromosomes Xq), TAOK1--NF2 (chromosomes 17q and chromosomes 22q), DENR--NF2 (chromosomes 12q and chromosomes 22q), NF2--MIF-AS1 (chromosomes 22q and chromosomes 22q), NF2--TTC28 (chromosomes 22q and chromosomes 22q) and NF2--SPATA13 (chromosomes 22q and chromosomes 13q). Notably, most fusion identified here are intrachromosomal fusion. To characterize the interchromosomal fusion, we then subset the fusions that were composed of two different chromosomes. A total of 91 fusions were shortlisted and more importantly, the fusion frequency (per Geta Base) of subtype 3 meningioma was highest of all subtypes (Figure 3B). Of the 91 interchromosomal fusions, PSPH is the most frequent fused gene and the most frequent fusion RP11-206L10.9 -- PSPH (35/91) presented in all subtypes (Figure 3C). We also uncovered subtype 3 specific fusions such as LPA--CAMSAP3 and MYO6--NSUN3, which were only found in subtype 3. More interestingly, almost all NF2 fusions were enriched in subtype 3, suggesting that NF2 was associated with meningioma progression, which is consistent with the previous study.

### 3.5. Different Types of Enriched Immune Cells between Subtypes Demonstrates the Disparate Level of Immune Cell Infiltration

**Figure 4:**
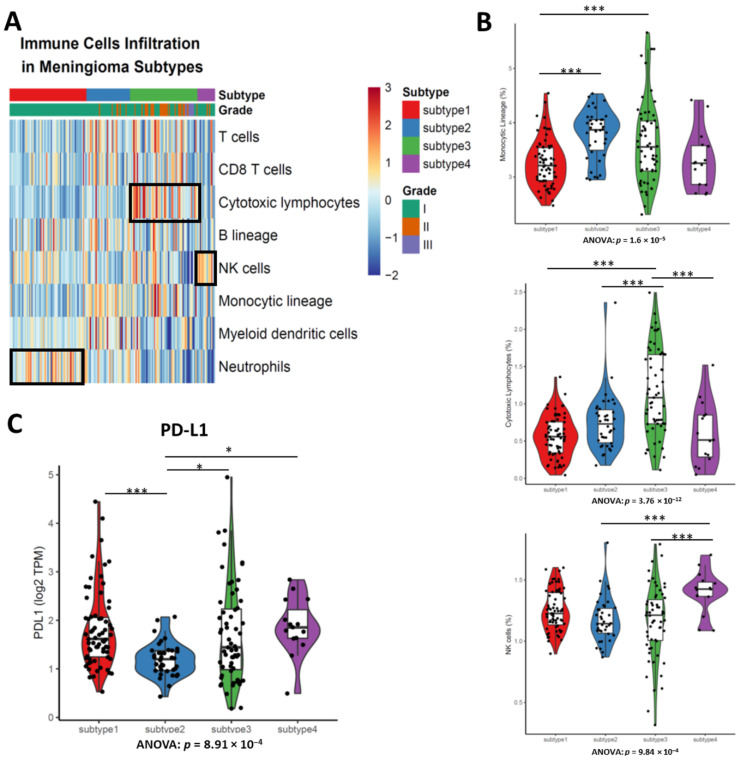
The levels of immune cell infiltration inferred by MCPcounter are distinct between subtypes of meningiomas. (**A**) Heatmap of immune cells infiltration highlights the enriched gene expression signature of distinct immune cells infiltration pattern between subtypes of meningiomas. Cytotoxic lymphocytes are enriched in subtype 3. Natural killer (NK) cells are enriched in subtype 4. Neutrophils are enriched in subtype 1. (**B**) Meningioma of subtype 3 has higher cytotoxic lymphocytes infiltration than others. Violin plots shows the immune cells infiltration between subtypes. (**C**) Subtypes have different PD-L1 expressions. ANOVA test, more than three groups comparison. Tukey’s HSD post hoc test. * *p* < 0.05; *** *p* < 0.001.

Since the immunotherapy including immune checkpoint inhibitor (PD-L1/PD-1 inhibitor) and CAR-T have demonstrated their antitumor efficacy in solid tumors, we next investigated the immune cell infiltration and PD-L1 expression in meningiomas. MCPcount was utilized for immune cells infiltration calculation. The heatmap of each immune cell lineage demonstrated the difference of immune cell response to subtype of meningiomas (Figure 4A). There was significant difference of cytotoxic lymphocytes infiltration between subtypes (ANOVA, *p* = 3.76 × 10^−12^), where subtype 3 had the highest cytotoxic lymphocytes infiltration of all meningioma subtypes (Figure 4B). We also observed more natural killer (NK) cells were likely to be presented in subtype 4 of meningioma, suggesting NK cell infiltration likely happen in one subtype of meningioma (Figure 4B).

Although a previous study revealed the PDL1 positive were rare in meningioma (4/58) and PDL1 expression is not associated with WHO grades [27], whether the transcripts of PDL1 was enriched in some subtypes remains unclear. The PDL1 expression in subtype 2 was significantly lower than the other three subtypes (ANOVA, *p* = 0.000891), suggesting patients with subtypes 1, 3 and 4 of meningioma likely benefit from immune checkpoint blockade (Figure 4C).

### 3.6. Random Forest (RF) Identifies Downregulated ALPL as the Feature Genes in Subtypes 3

**Figure 5:**
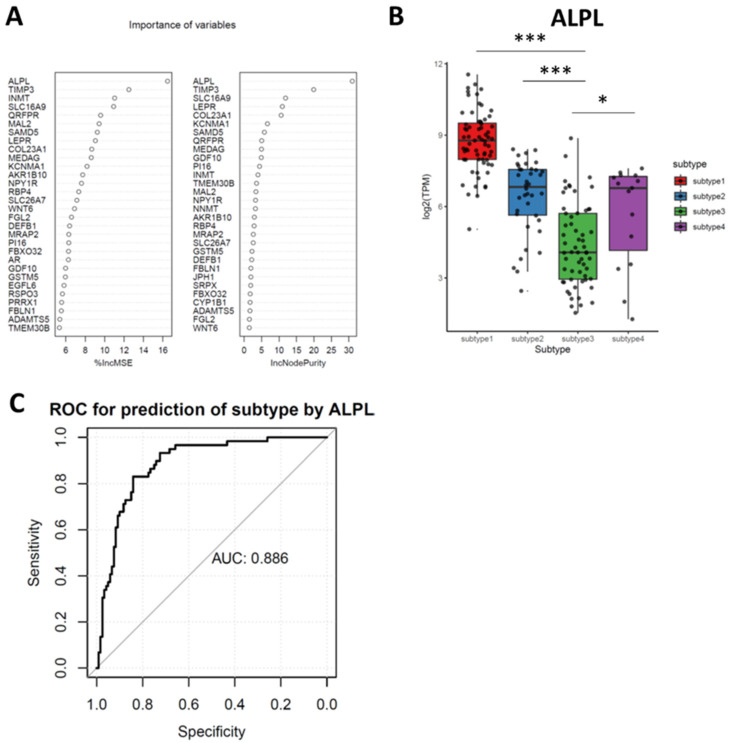
Random forest identifies Alkaline Phosphatase (ALPL) as the feature genes in subtypes 3. (**A**) Top 20 important genes (variables) are ranked by Random Forest model. (**B**) ALPL in subtype 3 is significantly lower than that in the other subtypes of meningioma (One-way ANOVA: *p* < 2 × 10^−16^; post hoc, Tukey’s HSD test; * *p* < 0.05; *** *p* < 0.001). (**C**) The receiver operation curve shows the discrimination of subtype 3 over other subtypes of meningioma by ALPL expression.

Our study identified subtype 3 was the most progressive meningioma subtypes, therefore, one biomarker differentiating subtype 3 meningioma from others could identify the risks of meningioma progression. A random forest (RF) algorithm was utilized for subtype 3 featured genes identification. RF calculated the importance of each gene (variable) to the subtyping (feature) and the top important genes were listed in Figure 5A. As noted, ALPL was identified as the top featured gene for subtype 3 meningioma by RF (Figure 5B and Figure S6). To further explore whether ALPL could be used for the predictive biomarker of meningioma progression, we performed the prediction capacity analysis by the receiver operation curve (ROC). The ROC displayed ALPL had a remarkable prediction ability with accuracy of 0.886 (Figure 5C).

### 3.7. Meningioma Progression Score (MPscore) Discriminates the Progression of Meningioma

**Figure 6:**
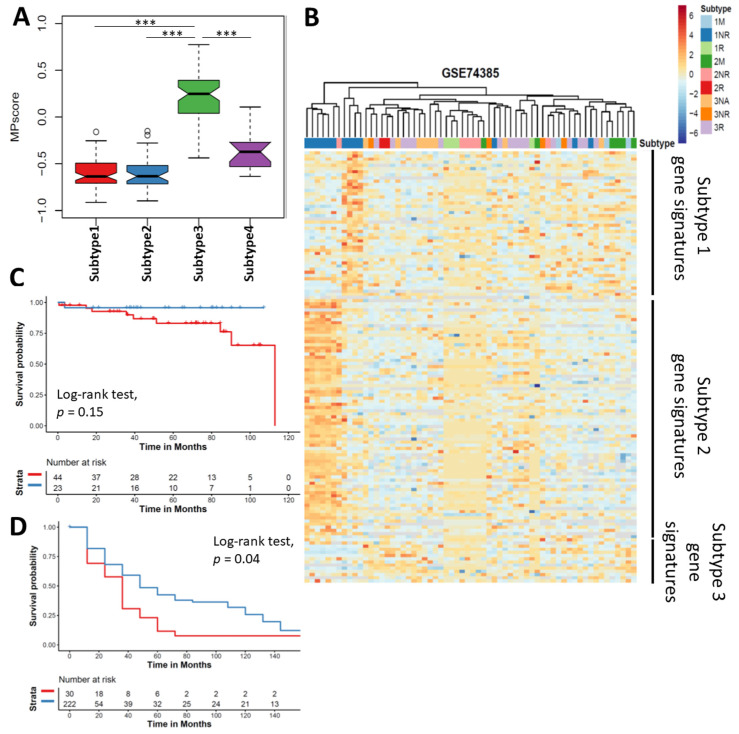
Validation of meningioma progression score. (**A**) Boxplot showing the MPscore in Subtype 3 was significantly higher than other subtypes. (**B**) Heatmap showing our clustering is also observed in validation cohort. Kaplan-Meier Curves showing the overall survival (**C**) and recurrent free survival (**D**) stratified by MPscore. Log-rank test was performed to examine the statistical difference. Red, high MPscore; blue, low MPscore. *** *p* < 0.001.

To generalize the discriminative capacity of the clustering by the progressive features and quantify the likelihood of progression in meningioma, we constructed a meningioma progression score (MPscore). As subtype 3 meningioma was identified as the progressive meningioma, we selected the significantly differentially expressed genes of subtype 3 as meningioma progressive signatures (Table S1). Based on the ssGSEA algorithm, we summarized the scores of subtype 3 signatures as the MPscore for given samples. Our MPscore of subtype 3 was significantly higher than other three subtypes, suggesting our MPscore was a suitable surrogate for subtype 3 and meningioma progression (Figure 6A). To validate the clinical utility of MPscore, we first utilized a microarray dataset (GSE74385) from a cohort containing a total of 62 meningiomas in grade I, II and III meningioma [6]. Unsupervised clustering of DEGs in these 62 meningioma samples revealed a clear separation of grade I nonrecurrent meningioma from recurrent or high-grade meningioma by our subtype gene signatures (Figure 6B), suggesting the clinical utility of our clustering. To validate the predictive utility of MPscore, we compared the MPscore between nonrecurrent and recurrent meningioma and found MPscore in metastatic or recurrent meningioma was significantly higher than that of non-recurrent meningioma (Figure S7). Then, we tested our MPscore in our own and another two independent cohorts of gene expression microarrays (Supplementary Methods, Table S4). All of them showed a significant higher MPscore was observed in the recurrent (or anaplastic) meningiomas as compared with nonrecurrent (or grade I) meningioma (*p* = 0.002376, *p* < 2.2 × 10^−16^ and *p* = 1.928 × 10^−5^) (Figure S7). To further demonstrate the prognostic ability of MPscore, we then examined the overall and recurrent free survival in meningioma. Although the overall survival of meningioma patients with low MPscore was not statistically better than that with high MPscore (*p* = 0.15), patients with low MPscore had a significantly better recurrent free survival (*p* = 0.04), suggesting MPscore could be a prognostic biomarker for meningioma recurrence (Figure 6C,D). Multivariable Cox proportion hazard regression model revealed MPscore was significantly associated with the recurrence free survival in meningioma (Table 2). Taken together, these results highlighted that our MPscore could surrogate the meningioma progression.

### 3.8. Small Molecules were Predicted to Target the Subtype 3 Meningioma

Lastly, we queried the Connectivity Map 2 (https://clue.io, accessed on 1 August 2020) to predict which compound was potentially the therapeutics for subtype 3 meningioma. We applied the differentially expressed genes of subtype 3 into the Connectivity Map 2 and 1309 compounds were screened. Of them, 16 compounds were identified as the potential therapeutic targets for subtype 3 meningioma (Table S5).

## 4. Discussion

Accumulating evidence has indicated meningioma is a heterogeneous tumor but the feature of clinical recurrent meningioma is still unclear. Through the consensus clustering of meningioma transcriptomes, we identified four mutually distinct subtypes of meningioma and all grade III and most grade II are clustered in subtype 3 of meningioma. This result suggests that the characterization of the differentially expressed genes in subtype 3 will uncover the biological features of meningioma progression (malignancy and recurrence). Meningioma in subtype 3 has distinct gene expression pattern to other subtypes, which confirms the heterogeneity of meningioma. Our analyses of noncoding RNAs and DNA methylation also further confirms the biological complexity of the tumorigenesis and progression in meningioma. As machine learning is widely used for biomarker discovery and potential therapeutic targets screening in cancer research [28,29] and random forest (RF)-based classification and featured variable identification has demonstrated the advantages of non-overfitting and robustness over the conventional differentially expressed gene analysis [30], we employed RF for integration of feature gene in subtype 3.

ALPL is identified as the top featured gene of all subtype 3 DEGs contributing to the features in subtype 3 of meningioma. The product of ALPL is a membrane bound glycosylated enzyme that broadly participating in phosphatase activity and alkaline phosphatase activity, therefore, the alteration of ALPL is found to be associated with hypophosphatasia and prostate cancer bone metastasis [31,32]. Our results suggest ALPL is likely a subtyping and recurrence diagnostic biomarker with a significant accuracy, which is consistent with the previous studies [33,34]. Although RF predicts TIMP3, INMT and SLC16A1 followed by ALPL as the important feature genes in subtype 3, the expressions of these three genes are not consistently lower or higher than all other subtypes of meningioma, which restricts their potent of being biomarkers for meningioma. The molecular pathway alterations caused by the reduced ALPL in meningioma is still needed to be investigated further though a few studies uncover the association of ALPL and NOTCH1 regulation in human epithelial cells and ALPL is one of the key hub genes in glioblastoma [35,36].

In order to identify the progressive subtype in meningioma, we constructed MPscore based on the featured genes in subtype 3. Our MPscore demonstrates a remarkably discriminative capacity for progressive meningioma in two independent cohorts. A few attempts have been made to explore the recurrence or progression related genes in meningioma. However, there is an inconsistence of meningioma progressive gene lists between studies [6,37,38,39]. Direct comparison between grade I and anaplastic or atypical meningioma unlikely provides the generic progressive genes because high-grade transformation has already occurred in grade I meningioma in early stage [40,41]. We construct a reference-based MPscore for the prediction of meningioma progression. We also validated our MPscore in three independent cohorts, highlighting the clinical utility of MPscore across different profiling sources (RNA seq or microarray). A total of 53 genes are included as the progressively phenotypic references in our study. In our MPscore gene reference, LEPR is another well-characterized prognostic biomarker and independent predictive biomarker for meningioma. Loss of function of LEPR is associated with the elevated leptin levels and obesity, showing its participating in adipose biogenesis [42]. Although the biological regulation of LEPR in meningioma is still unclear, a few observative studies uncover patients with meningioma are prone to be obese [43,44]. Our clustering is consistent with the main findings of previous studies, which also suggests the robustness of our analyses. Patel et al. find a subgroup of meningioma has a shorter recurrence-free survival by clustering of WHO grade I and II meningioma while the other two subgroups have relatively low risks of recurrence. Our results of clustering echo their findings where a few grade I meningiomas (subtype 3) have similar transcriptome pattern to the recurrent meningioma while most grade I meningiomas are clustered into two distinct subtypes (subtype 1 and 2). Notably, our results further reveal these grade I meningiomas that likely recur share similar transcriptome patterns with grade III meningioma, confirming that progressive transformation happens in grade I meningioma. Another clustering of WHO grade I meningioma indicates there are five subgroups in meningioma but four of five subgroups are enriched in the WNT pathway. In our analyses, WNT-pathway-related genes such as DKK2 are involved in subtype 1 signature gene. Together with previous studies, the clustering by transcriptomes highlights the heterogeneity and the genetic variation in meningioma and subtyping of meningioma enable us to identify the meningioma with high risk of recurrence.

One previous study found some novel gene fusions involved in NF2, the most common mutation in meningioma. However, the NF2 gene fusion-based biomarker for the prediction of meningioma progression or recurrence might not be reliable in clinical practice as radiation therapy could induce new NF2 mutations or structural variants in meningioma [10,20]. Patients may not benefit from these biomarkers targeting postradiation NF2 gene fusions as these NF2 genes do not predict the risk of recurrence in the initial diagnosis of meningioma. Although our study fails to identify the subtype or recurrence specific biomarkers, we discovered that gene fusion with EYA1 is the most common fusion across all subtypes of meningioma, indicating EYA1 fusion is likely an early event in tumorigenesis in meningioma. EYA1 is a protein phosphatase and a transcriptional coactivator for SIX1 that regulates gene expression and cellular proliferation. In meningioma, EYA1 is also a key molecule by regulating the cell viability and cell cycle [45]. Compared with other types of brain tumor, meningioma has significantly higher expression of EYA1. Our results support the critical role of EYA1 in meningioma though further investigation is required to validate altered activated pathways caused by EYA1 fusion. These results shed a light on the tumor formation and potential therapeutic targets for meningioma.

The four subtypes of meningioma we identified also have different immune cell infiltration and PD-L1 expression. Previous small-scale studies display T cells and B cells infiltrated in meningiomas are antigen-experienced and monocytes are also present in meningioma, indicating the feasibility of immunotherapy application in meningioma [46,47]. Our results show that a broad spectrum of immune cells infiltration in meningioma, and moreover, we show monocytic lineage is the most predominant of all immune cell types. Of all subtypes, subtype 1 has the least immune cells (including monocytes, T cells and B cells) infiltration. That is likely because all subtype 1 meningiomas are benign and composed from WHO grade I meningioma. As noted, subtype 3 has the highest cytotoxic T cells infiltration of all subtypes, suggesting the enhanced T cells could be a potential therapeutic for patients in subtype 3 while NK-cell-based therapeutics may benefit patients in subtype 4 [48]. Our analysis of PD-L1 in meningioma is partially consistent with previous study where higher grade meningioma has higher levels of PD-L1 [45]. Our results show the levels of PD-L1 in subtype 3 and 4 are higher than that in subtype 2. However, we also found subtype 1 composed from WHO grade I meningioma has relatively higher level of PD-L1. We presume there probably is a post-transcriptional modification or regulation of PD-L1 in meningioma so it is rarely detected by antibody-based immunohistochemistry [27,45,49,50].

We noticed there are a few limitations in this study. Firstly, due to the accessibility to the materials and datasets, we do not crossvalidate our subtype clustering with the clustering by DNA methylation and mutation information. A comprehensive landscape of meningioma integrating mutation, structural variant, DNA methylation, RNA (mRNA and ncRNA) transcripts and proteomics will help us understand the biological behaviors of meningioma recurrence. Histopathological analyses of immune cells infiltration and cell cycle markers will also complement the microenvironment landscape of meningioma. Secondly, although we demonstrated that grade II meningiomas is likely presented in Subtype 3 and Subtype 3 meningiomas usually have a high expression level of mitotic genes, the association of biological subtypes with histopathological types remains unclear [51]. More studies on the distinct biological features within different histopathological types are under investigation. Thirdly, the prediction capacity of ALPL for meningioma recurrence needs to be validated externally. In this study, the high-grade meningioma surrogates the recurrent meningioma, which might be a selection bias. Therefore, prior to the clinical application, the assay designed for ALPL also should be elaborately tested for clinical utility in a large perspective cohort where meningioma patients developing to recurrence are recruited.

## 5. Conclusions

Our study provides the landscape of transcriptome in meningioma and identifies the recurrence of relevant subtypes of meningioma (subtype 3 meningioma) by consensus clustering. Loss of chromosome 1q with NF2 mutation or chromosome 22q loss is one of the genetic features in subtype 3. Hypermethylated CpG loci of the promoter areas spread the subtype 3 meningioma. Enhanced T cells therapy is likely the most promising immunotherapy for meningioma recurrence. Reference-based MPscore is potentially a predictive and prognostic biomarker for the recurrence in meningioma.

## Figures and Tables

**Table 1:** Characteristics of each clustering subtype in this study.

Items	Subtype 1	Subtype 2	Subtype 3	Subtype 4
Age (median, 25–75% quantile)	61, 47.5–68	61, 50–66	61,49.5–72	61, 58–68.5
Gender (Male:Female)	53:12	24:14	20:27	10:4
WHO Grade				
I	67	32	28	13
II	0	6	24	2
III	0	0	7	0

**Table 2:** Multivariable Cox proportion regression analysis.

Variables	Sub-Variables	Coefficient	Lower 95% CI	Upper 95% CI	*p* Value
MPscore		1.63762	2.00798	13.1721	0.000643
Location					
	Convexity	−0.19415	0.18135	3.7398	0.801446
	Falx	−0.44845	0.13599	2.9991	0.569863
	Intraventricular	0.59538	0.25013	13.1514	0.555854
	Olfactory groove	0.45279	0.32038	7.7202	0.576995
	Optic nerve	−0.50801	0.08373	4.3237	0.613643
	Parasagittal	−0.0126	0.2065	4.7222	0.987409
	Posterior fossa	0.55699	0.37301	8.1672	0.479293
	Sphenoid wing	−0.22505	0.17501	3.643	0.771353
	Supresellar	−1.27342	0.05222	1.5	0.137122
Gender		−0.34111	0.40969	1.2338	0.225178
MIB-1		−0.06811	0.87729	0.9947	0.033544

## Data Availability

The codes for this study are available from https://github.com/mackaay/MPscore. Raw RNA seq data was publicly available in Gene Expression Omnibus (accession GSE85135 and GSE136661). The analyzed datasets during the current study available from the corresponding author on reasonable request.

## Supplementary Materials

The following are available online at https://www.mdpi.com/2072-6694/13/5/1113/s1, Table S1: The differentially expressed gene (DEG) between subtypes, Table S2: The differentially expressed lncRNA between subtypes, Table S3: The fusion genes are identified in this study, Table S4: The characteristics of three validation cohorts, Table S5. Compounds were significantly predicted as the therapeutic targets for subtype 3 meningioma, Figure S1: PCA identifies the heterogeneity of meningiomas after batch effect correction, Figure S2: Meningiomas are clustered into four subtypes by the transcriptomes. The cumulative distribution function (CDF) curve (A) and the changed area under CDF curve (B) suggests k = 4 is the best number of subtype for clustering. (C), SigClust p-values for all pair wise comparisons of clusters. (D), the consensus clustering matrix plot showing the subtypes of meningioma when consensus k = 3 and 5, Figure S3: The levels of stemness indexes for meningiomas are distinct between WHO grades (left) and subtypes (right). Stemness Indexes in grade I meningioma is significantly lower than high grade meningioma. One-way ANOVA test for multiple group comparison; post hoc, Tukey’s HSD. * *p* < 0.05; ** *p* < 0.01; *** *p* < 0.001, Figure S4: Hypermethylated DNA is mostly observed in the subtype 3 meningioma. (A), typical CNA of subtype 1 (top panel), 2 (middle panel) and 3 (bottom panel) of meningioma. (B), the PCA plot showing the top 2000 variance of DNA methylation levels of the DNA methylation cohort. C, the weheatmap showing the CpG loci signatures of each subtype. D, the boxplot showing that the subtype 3 meningioma had the significantly highest methylation level of all the subtypes, Figure S5: The gene fusion in each subtype of meningioma. (A), the gene fusion frequency between subtypes. ANOVA test, *p* = 0.13. (B), Circus plots of subtype 1 (upper), 2 (bottom left) and 4 (bottom right) meningioma, Figure S6: Three genes are potentially the biomarker for subtype 3 of meningioma. (A), the expression of TIMP3, INMT and SLC16A1 between subtypes. (B), the ROCs of TIMP3, INMT and SLC16A for identification of subtype 3 against other subtypes. One-way ANOVA test for multiple group comparison; post hoc, Tukey’s HSD. * *p* < 0.05; ** *p* < 0.01; *** *p* < 0.001, Figure S7: The clinical utility of MPscore is validated in four independent cohorts. 1M, grade I metastatic meningioma; 1NR, grade I non-recurrent meningioma; 1R, grade I recurrent menigioma; 2M, grade II metastatic meningioma; 2NR, grade II non-recurrent meningioma; 2R, grade II recurrent meningioma; 3NA, grade III meningioma; 3NR, grade III non-recurrent meningoma; 3R, grade III recurrent meningioma. The statistical significance was performed by Student’s *t* test. * *p* < 0.05; ** *p* < 0.01; *** *p* < 0.001.
