# Supplementary material for: MPscore: A Novel Predictive and Prognostic Scoring for Progressive Meningioma
Source: Cancers (Basel). 2021 Mar 5;13(5):1113. doi: 10.3390/cancers13051113 (PMC7961759; doi:10.3390/cancers13051113)
Supplement: Supplementary file 1 [file cancers-13-01113-s001.zip › cancers-1100042-supplementray/cancers-1100042-supplementray.pdf]

cancers

MDPI

# Supplementary Methods: MPscore: A Novel Predictive and Prognostic Scoring for Progressive Meningioma

**Feili Liu, Jin Qian and Chenkai Ma**

## Tissue Samples and Microarray Analysis

Six meningioma samples (3 fibroblastic meningioma and 3 anaplastic meningioma) from our previous study were utilized for MPscore validation [1]. Tissue samples were stored in −80 °C freezer after surgical operation. Histopathological grade and types were determined by H&E staining. Total RNA was extracted from these meningioma tissue samples by Trizol. Agilent Bioanalyzer 2100 was used for RNA quality and yield assessment. cDNA was constructed by two rounds synthesis where total RNA was revers-transcribed by One-Cycle cDNA Synthesis kit (Affymetrix) in the first-round. After the second-round amplification by the GeneChip IVT Labeling Kit (Affymetrix), the labelled product was subject to hybridized to the Affymetrix GeneChip Human U133 Pluss 2.0 Array. Microarray data was modelled by the Robust Multichip Average approach.

For another three independent cohorts (GSE74385, GSE16181 and GSE16581), the datasets were downloaded queried by the R package "GEOquery" [2,3]. The details of each dataset were listed in the Supplementary table 5.

## MPscore Validation

MPscore was calculated for each sample from our cohort and the other two datasets. Differentially expressed genes in subtype 3 were considered as the signatures of meningioma progression score (MPscore). The MPscore was the sum of the difference of ssGSEA-predicted scores from up- or down-regulated gene list. Due to the batch effect, the MPscore calculation was performed per cohort. The missing value was replaced by the median value of each sample.

$$MPscore = \sum ssGSEA(upregulated\ signatures) - ssGSEA(downregulated\ signatures)$$

## Survival Analysis

The overall survival and recurrent free survival analyses was performed at GSE16581 and GSE16181 dataset, respectively. The best cut-off value of MPscore for survival stratification was determined by the R package "survminer". Kaplan-Meier Curves using Log-rank test were conducted to investigate the survival difference between groups with different MPscore. Cox proportional hazard regression model was applied to identify the association of multiple variables with patients' survivals.

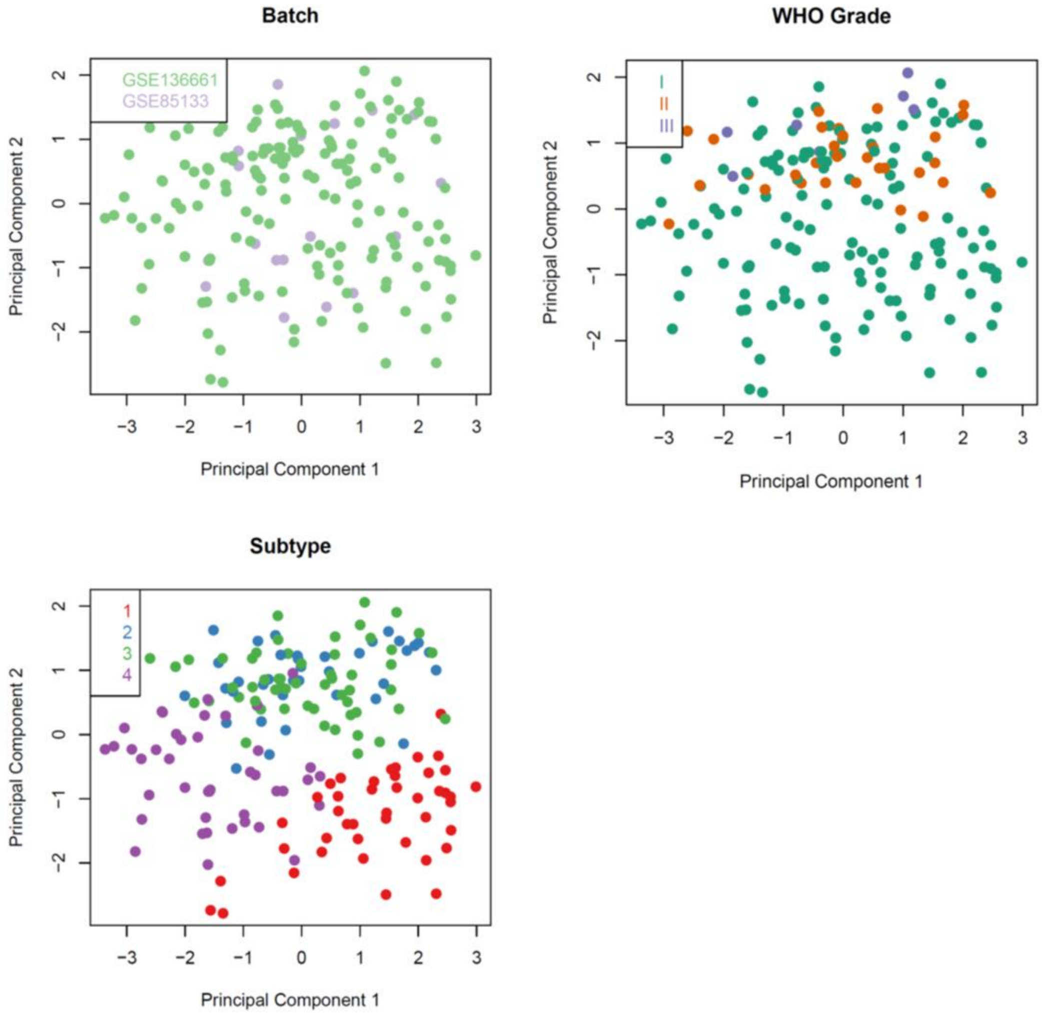


**Figure S1.** PCA identifies the heterogeneity of meningiomas after batch effect correction.

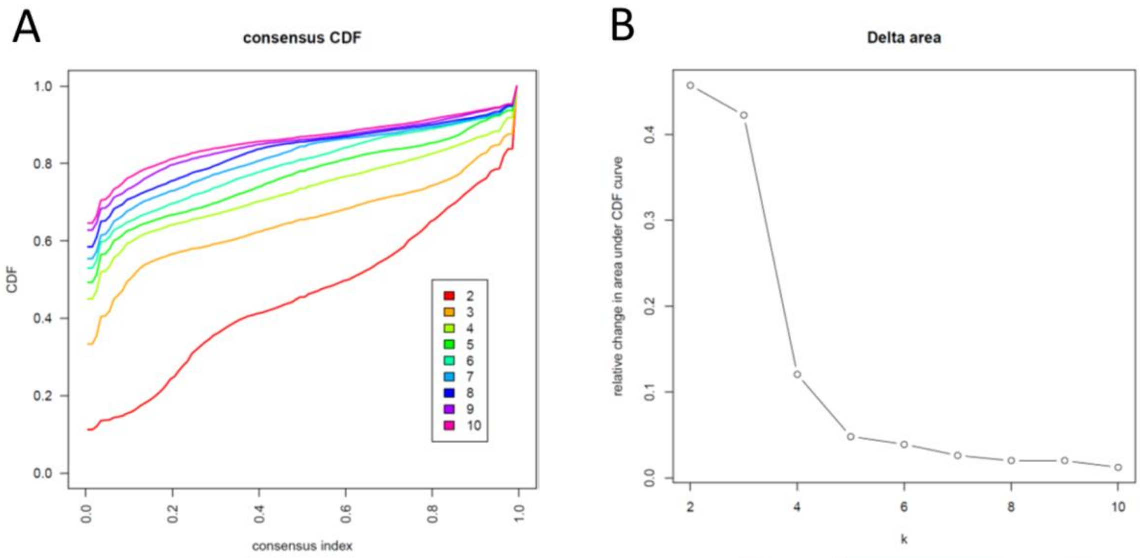


C

| | Subtype1 | Subtype2 | Subtype3 |
|---|---|---|---|
| **Subtype2** | p = 0 | | |
| **Subtype3** | p = 0 | p = 0 | |
| **Subtype4** | p = 0 | p = 0 | p = 0 |

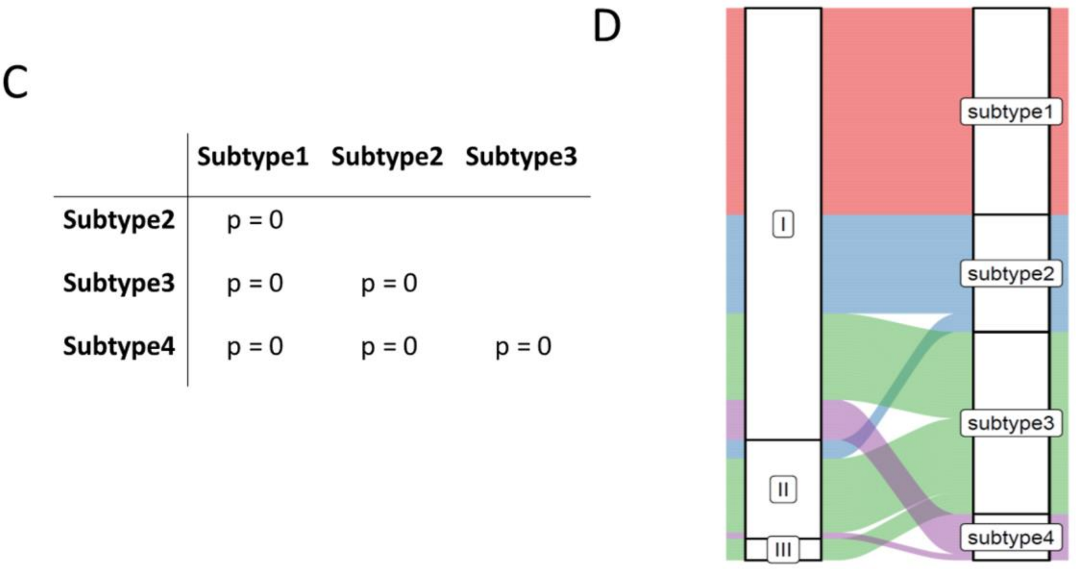


**Figure S2.** Meningiomas are clustered into four subtypes by the transcriptomes. The cumulative distribution function (CDF) curve (A) and the changed area under CDF curve (B) suggests k = 4 is the best number of subtype for clustering. (C), SigClust p-values for all pair wise comparisons of clusters. (D), the consensus clustering matrix plot showing the subtypes of meningioma when consensus k = 3 and 5.

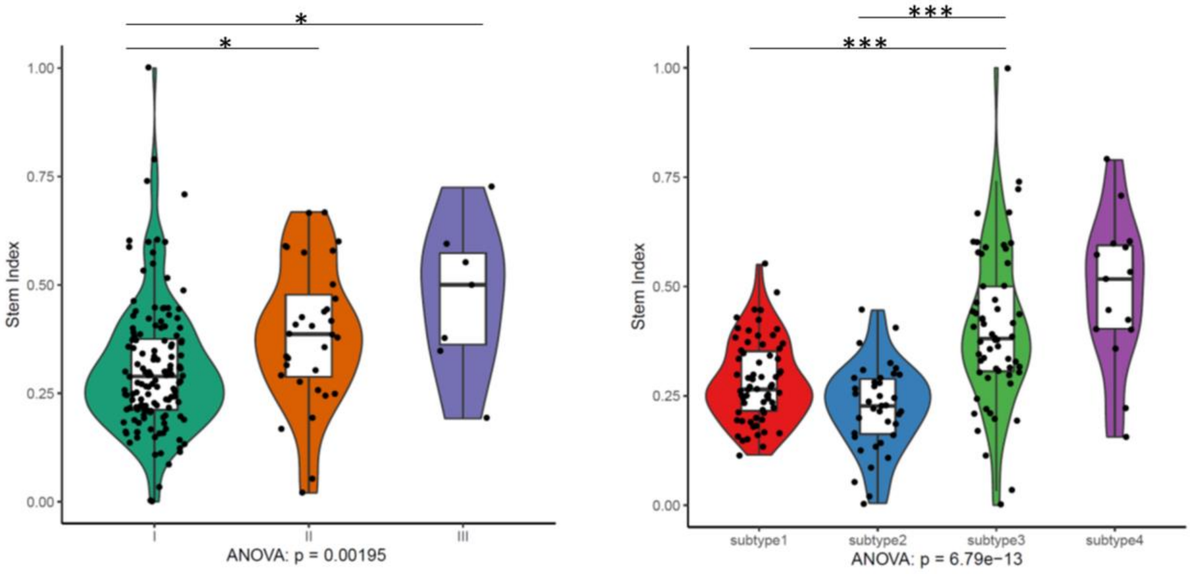


**Figure S3.** The levels of stemness indexes for meningiomas are distinct between WHO grades (left) and subtypes (right). Stemness Indexes in grade I meningioma is significantly lower than high grade meningioma. One-way ANOVA test for multiple group comparison; post hoc, Tukey's HSD. * $p < 0.05$; ** $p < 0.01$; *** $p < 0.001$.

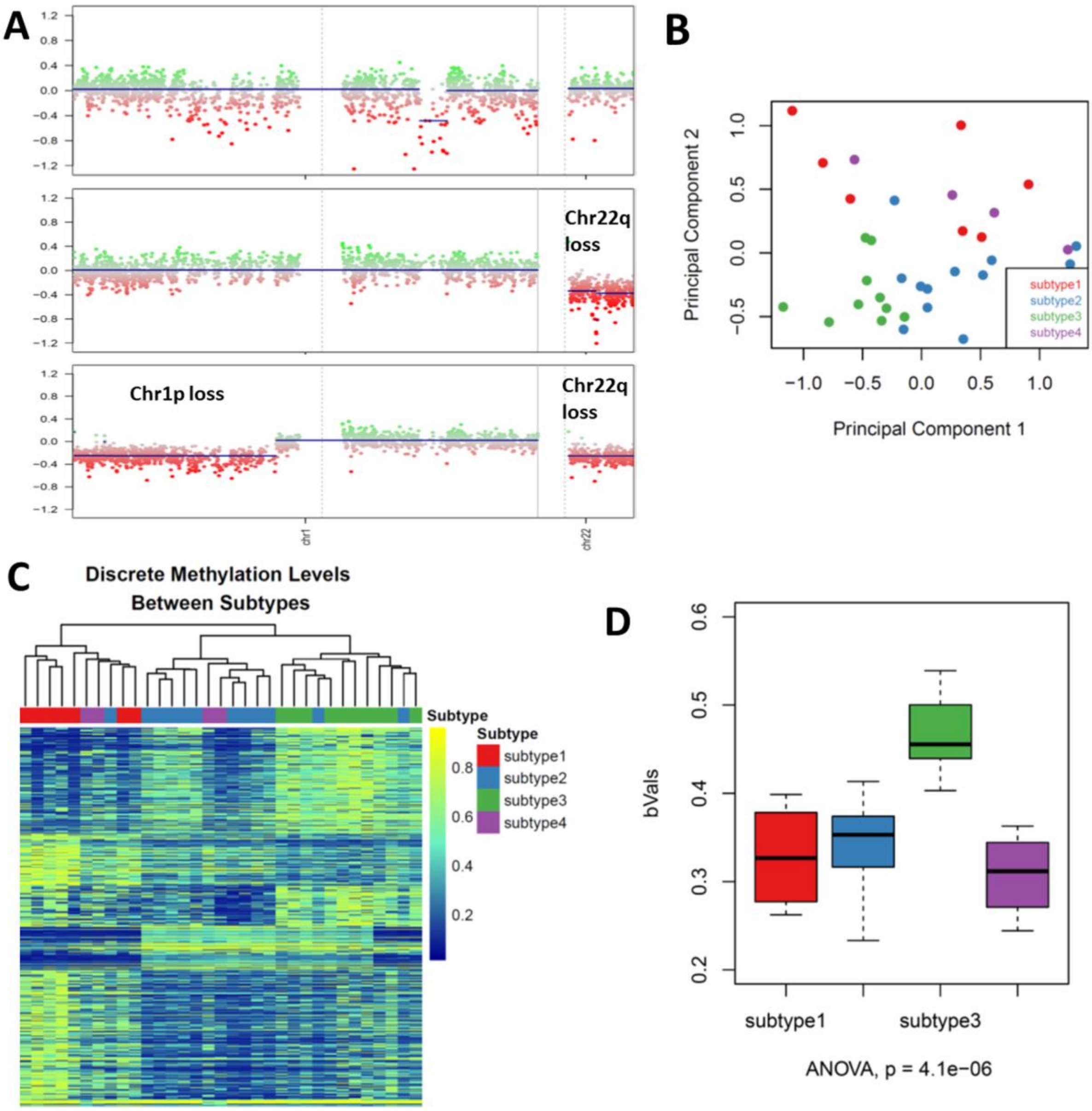


**Figure S4.** Hypermethylated DNA is mostly observed in the subtype 3 meningioma. (A), typical CNA of subtype 1 (top panel), 2 (middle panel) and 3 (bottom panel) of meningioma. (B), the PCA plot showing the top 2000 variance of DNA methylation levels of the DNA methylation cohort. C, the weheatmap showing the CpG loci signatures of each subtype. D, the boxplot showing that the subtype 3 meningioma had the significantly highest methylation level of all the subtypes.

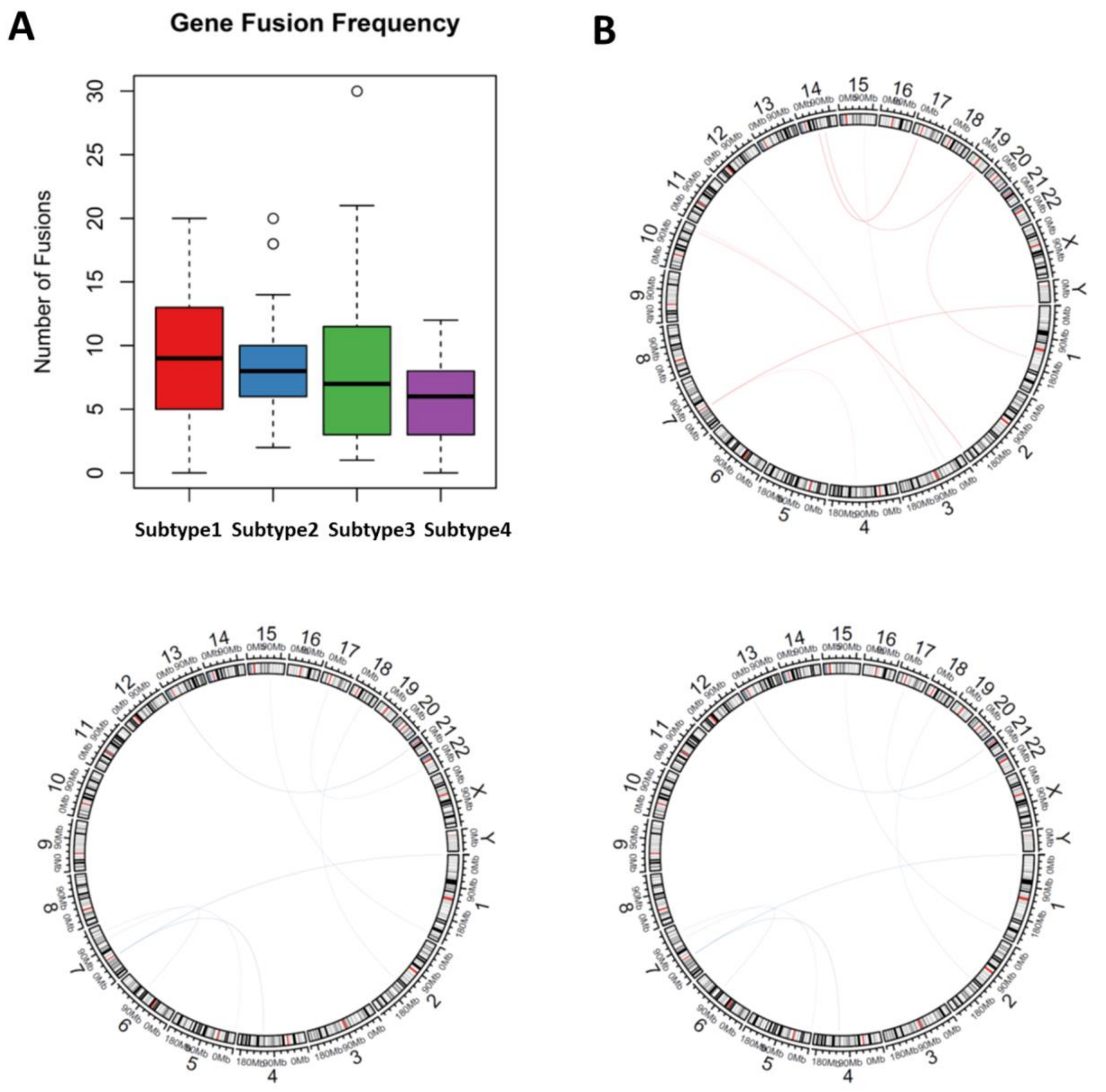


**Figure S5.** The gene fusion in each subtype of meningioma. (A), the gene fusion frequency between subtypes. ANOVA test, $p = 0.13$. (B), Circus plots of subtype 1 (upper), 2 (bottom left) and 4 (bottom right) meningioma.

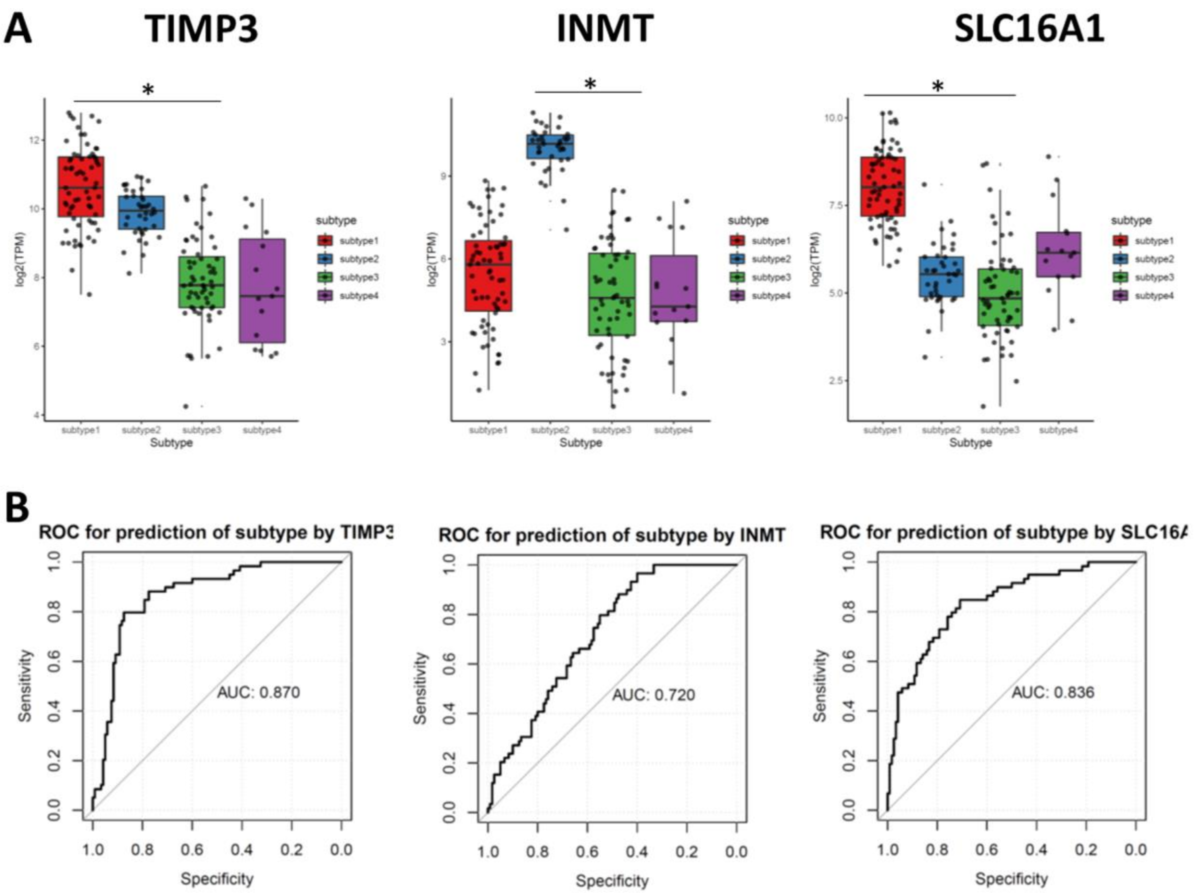


**Figure S6.** Three genes are potentially the biomarker for subtype 3 of meningioma. (A), the expression of TIMP3, INMT and SLC16A1 between subtypes. (B), the ROCs of TIMP3, INMT and SLC16A for identification of subtype 3 against other subtypes. One-way ANOVA test for multiple group comparison; post hoc, Tukey's HSD. * $p < 0.05$; ** $p < 0.01$; *** $p < 0.001$.

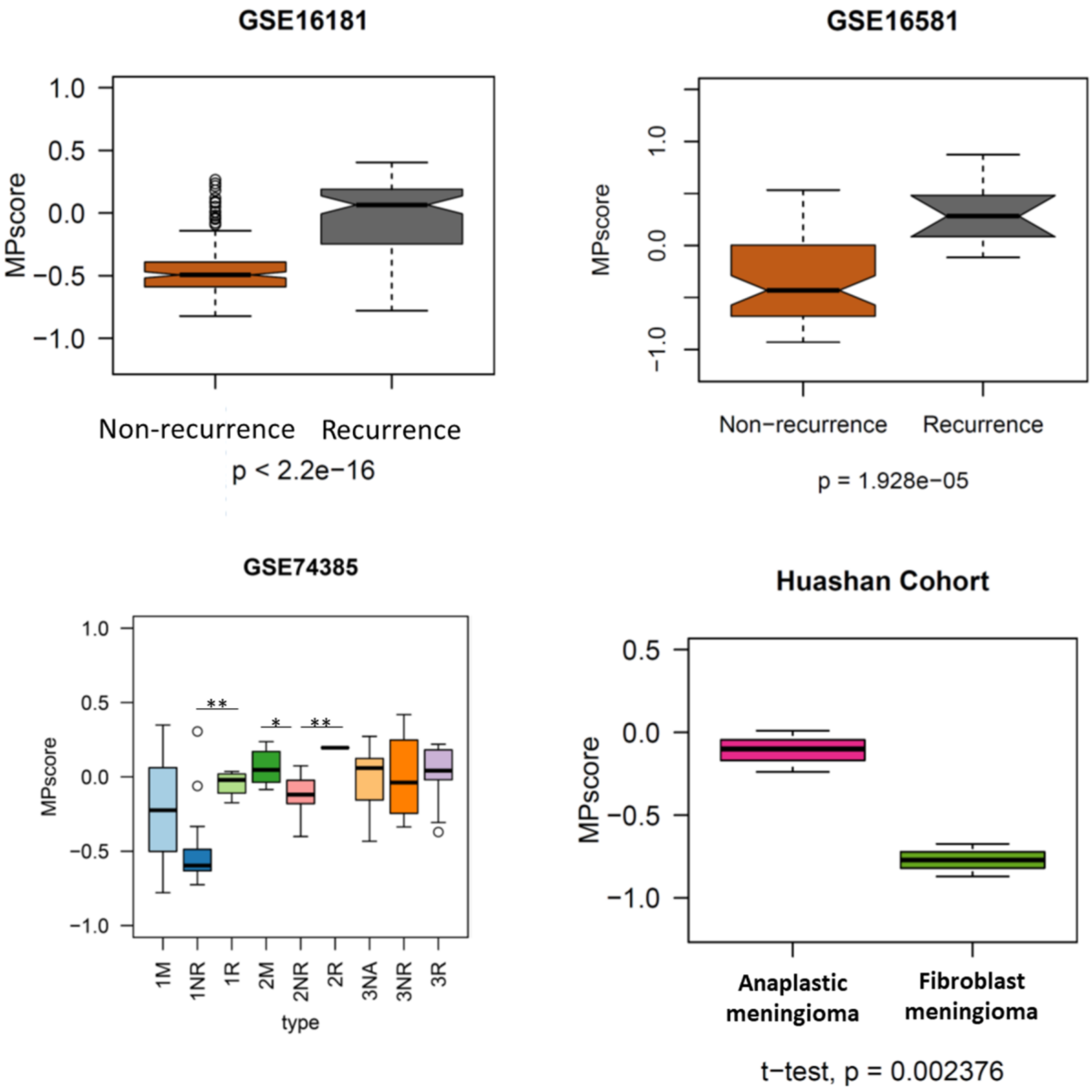


**Figure S7.** The clinical utility of MPscore is validated in four independent cohorts. 1M, grade I metastatic meningioma; 1NR, grade I non-recurrent meningioma; 1R, grade I recurrent menigioma; 2M, grade II metastatic meningioma; 2NR, grade II non-recurrent meningioma; 2R, grade II recurrent meningioma; 3NA, grade III meningioma; 3NR, grade III non-recurrent meningoma; 3R, grade III recurrent meningioma. The statistical significance was performed by Student's *t* test. * $p < 0.05$; ** $p < 0.01$; *** $p < 0.001$.

## References


1. Wang, X.; Gong, Y.; Wang, D.; Xie, Q.; Zheng, M.; Zhou, Y.; Li, Q.; Yang, Z.; Tang, H.; Li, Y.; et al. Analysis of gene expression profiling in meningioma: Deregulated signaling pathways associated with meningioma and EGFL6 overexpression in benign meningioma tissue and serum. *PLoS ONE* **2012**, *7*, e52707.
2. Lee, Y.; Liu, J.; Patel, S.; Cloughesy, T.; Lai, A.; Farooqi, H.; Seligson, D.; Dong, J.; Liau, L.; Becker, D.; et al. Genomic landscape of meningiomas. *Brain Pathol.* **2010**, *20*, 751–762.
3. Schmidt, M.; Mock, A.; Jungk, C.; Sahm, F.; Ull, A.T.; Warta, R.; Lamszus, K.; Gousias, K.; Ketter, R.; Roesch, S.; et al. Transcriptomic analysis of aggressive meningiomas identifies PTTG1 and LEPR as prognostic biomarkers independent of WHO grade. *Oncotarget.* **2016**, *7*, 14551–14568.
